# Exploring the bases for a mixed reality stroke rehabilitation system, Part II: Design of Interactive Feedback for upper limb rehabilitation

**DOI:** 10.1186/1743-0003-8-54

**Published:** 2011-09-08

**Authors:** Nicole Lehrer, Yinpeng Chen, Margaret Duff, Steven L Wolf, Thanassis Rikakis

**Affiliations:** 1 School of Arts, Media and Engineering, Arizona State University, Tempe, USA; 2 Department of Bioengineering, Arizona State University, Tempe, USA; 3 Department of Rehabilitation Medicine, Emory University, Atlanta, USA

## Abstract

**Background:**

Few existing interactive rehabilitation systems can effectively communicate multiple aspects of movement performance simultaneously, in a manner that appropriately adapts across various training scenarios. In order to address the need for such systems within stroke rehabilitation training, a unified approach for designing interactive systems for upper limb rehabilitation of stroke survivors has been developed and applied for the implementation of an Adaptive Mixed Reality Rehabilitation (AMRR) System.

**Results:**

The AMRR system provides computational evaluation and multimedia feedback for the upper limb rehabilitation of stroke survivors. A participant's movements are tracked by motion capture technology and evaluated by computational means. The resulting data are used to generate interactive media-based feedback that communicates to the participant detailed, intuitive evaluations of his performance. This article describes how the AMRR system's interactive feedback is designed to address specific movement challenges faced by stroke survivors. Multimedia examples are provided to illustrate each feedback component. Supportive data are provided for three participants of varying impairment levels to demonstrate the system's ability to train both targeted and integrated aspects of movement.

**Conclusions:**

The AMRR system supports training of multiple movement aspects together or in isolation, within adaptable sequences, through cohesive feedback that is based on formalized compositional design principles. From preliminary analysis of the data, we infer that the system's ability to train multiple foci together or in isolation in adaptable sequences, utilizing appropriately designed feedback, can lead to functional improvement. The evaluation and feedback frameworks established within the AMRR system will be applied to the development of a novel home-based system to provide an engaging yet low-cost extension of training for longer periods of time.

## Background

Sensorimotor rehabilitation can be effective in reducing motor impairment when engaging the user in repetitive task training [1]. Virtual realities (exclusively digital) and mixed realities (combining digital and physical elements) can provide augmented feedback on movement performance for sensorimotor rehabilitation [2-8]. Several types of augmented feedback environments may be used in conjunction with task oriented training. Some virtual reality environments for upper limb rehabilitation have been categorized as "game-like" because the user accomplishes tasks in the context of a game, while some are described as "teacher-animation", in which the user is directly guided throughout his movement [9]. Among the teacher-animation environments for upper limb rehabilitation, several provide a three-dimensional representation of a hand or arm controlled by the user, which relate feedback to action by directly representing the user's experience in physical reality. Some applications, in contrast, use simple abstract environments (e.g., mapping hand movement to moving a cursor) to avoid providing potentially extraneous, overwhelming or confusing information. However, because functional tasks require knowledge and coordination of several parameters by the mover, an excessive reduction in complexity of action-related information may impede functional rehabilitation [10,11]. Augmented feedback for rehabilitation can best leverage motor learning principles if it allows the participant to focus on individual aspects of movement in the context of other key aspects of the trained movement. Therefore feedback should promote understanding of the relationships among multiple movement components.

Feedback used for rehabilitation training must also be adaptable in design, allowing for changes in training intensity and focus. Yet few existing augmented reality rehabilitation environments effectively communicate multiple aspects of movement performance simultaneously, or furthermore, do so in a manner that is adaptable and generalizes across multiple training scenarios.

In our companion paper, Lehrer et al present a methodology for developing interactive systems for stroke rehabilitation that allow for adaptive, integrated training of multiple movement aspects [12]. While the methodology may be generalized to different types of movement training within stroke rehabilitation, this paper applies the methodology to interactive reach and grasp training as exemplified in the Adaptive Mixed Reality Rehabilitation (AMRR) System.

We now provide an overview of the AMRR system and participant experience, followed by a more detailed discussion of the applied design methodology within the system's implementation. An action representation for reach and grasp training is presented with accompanying methods for quantifying the representation's kinematic features, which allow for measurable evaluation of performance and generation of media-based feedback. Descriptions of how the AMRR feedback addresses specific movement challenges are then provided, with corresponding multimedia examples. An overview of the system's adaptation of the feedback and training environments demonstrates how AMRR training can be customized for each stroke survivor. Finally supportive data from three participant cases are presented to demonstrate the system's ability to promote integrated improvement of several movement features. Correlations between performance improvements in trials following the presence of observable feedback are also presented in support of the feedback design's efficacy in promoting self-assessment by the participant. A full results paper evaluating the use of AMRR therapy in comparison to traditional therapy will be provided in a forthcoming paper after the conclusion of a clinical study currently underway. The main intent of this paper is to provide a detailed description of the implemented methodology for interactive feedback within the AMRR system based on principles established in [12].

## Results

### System Overview

**Figure 1:**
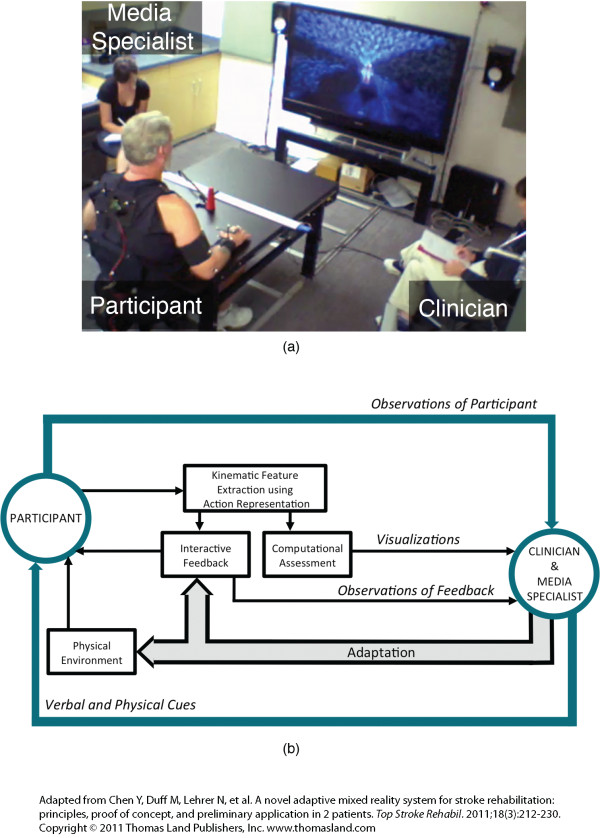
**AMRR system overview**. The system captures a participant's movement and extracts key kinematic features identified within the action representation. This kinematic data is used for computational assessment and generates the interactive feedback. Based on observation and the computational assessment, the clinician may adapt the system.

The Adaptive Mixed Reality Rehabilitation (AMRR) system provides detailed evaluation information and interactive audiovisual feedback on the performance of a reach and grasp task for the upper extremity rehabilitation of stroke survivors. See additional file 1: AMRR system demonstration to view the AMRR system in use. Figure 1 presents an overview of the AMRR system's components. The system uses motion capture to track a participant's movement throughout a reach and grasp task and extracts the key kinematic features of the action representation described in Lehrer et al [12]. These kinematic features are used for computational evaluation of the participant's performance, which can assist a clinician's assessment through summary visualizations. The kinematic features also generate the interactive feedback experienced by the participant. The term adaptive in this context refers to the ability of the therapist to adjust components of the system (e.g. feedback or physical components of the system) to accommodate the participant throughout training. The clinician may also use physical or verbal cues to further provide guidance when the feedback is not clearly understood by the participant.

**Figure 2:**
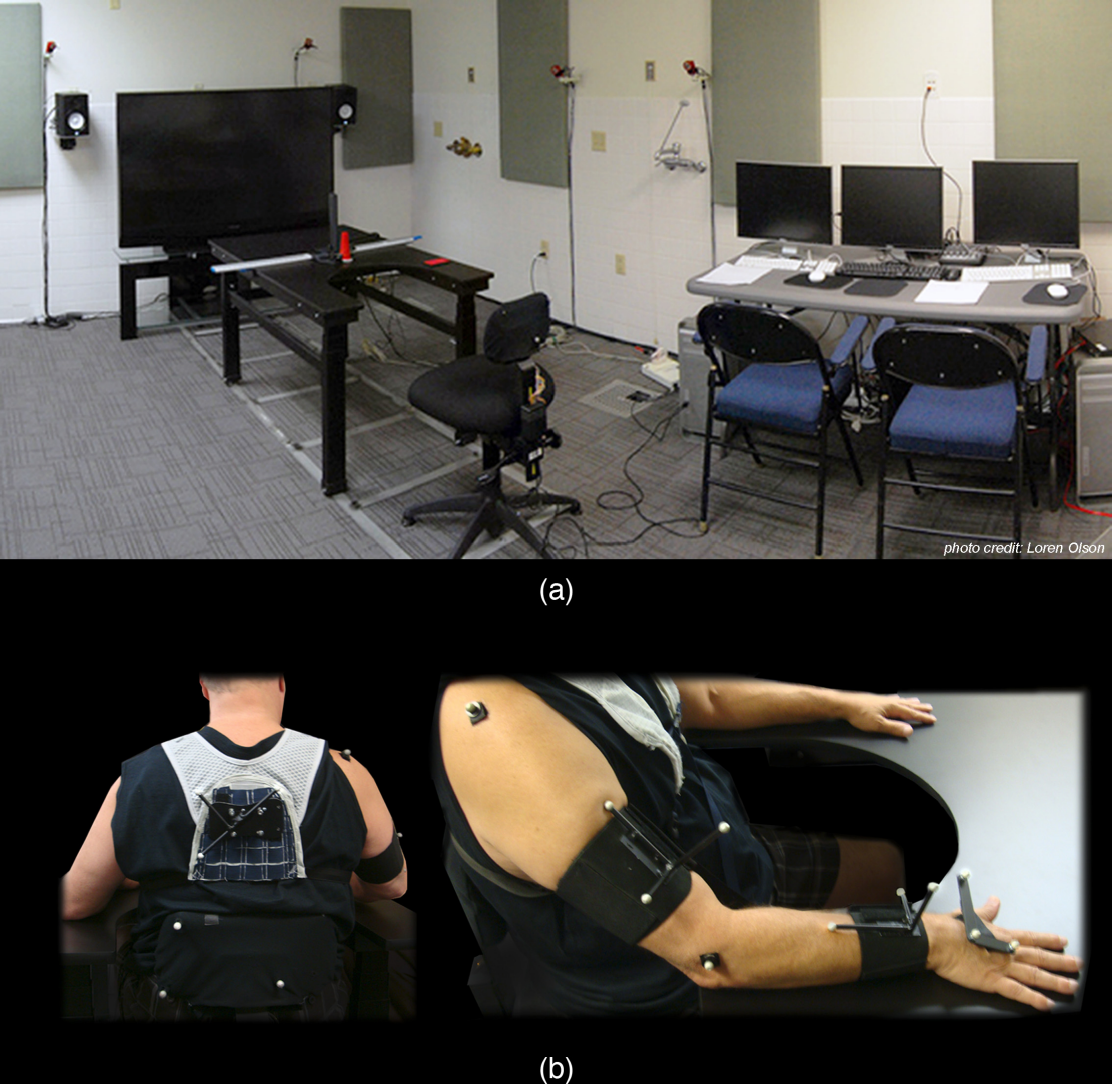
**System Apparatus and participant marker placement**. The system uses 11 Opti-Track cameras (not all cameras shown) to track 14 reflective markers worn by the participant on his back, shoulder blade, acromium process, lateral epicondyle, and the top of his hand, as well as 3 additional markers on the chair.

Figure 2(a) depicts an overview of the AMRR system apparatus. The system uses 11 Opti-Track FLEX:V100 R2 cameras to track 14 reflective markers, shown in Figure 2(b), worn by the participant on his back, shoulder blade, acromium process, lateral epicondyle, and the top of his hand, with 3 additional markers on the chair. The system tracks the participant's movement at a rate of 100 Hz, with a spatial resolution of 3.5 - 5.5 mm. Interaction with target objects on the table is sensed though a capacitive touch sensor within a button object (used in reach-to-touch tasks) and an array of force sensing resistors (FSRs) on a cone object (used in reach-to-grasp tasks). Embedded FSRs within the chair monitor the extent of support provided for the participant's torso and back. Currently, sensor data collected by the button object is used in real-time interaction to determine if the task was completed, while cone FSR data is being collected to inform the development of objects that provide feedback on grasping performance. FSR data collected by the chair is being used to develop a smart chair for monitoring torso compensation within a home-based training system.

The system is used by stroke survivors presenting clinical symptoms consistent with left-sided motor area lesions resulting in right-sided hemiparesis, who were right hand dominant prior to stroke. Each participant must demonstrate active range of motion in the right arm, with the following minimum movement thresholds to ensure they can complete the reaching task: shoulder flexion of at least 45°, elbow ROM of at least 30°-90°, forearm pronation or supination of at least 20°, wrist extension of at least 20°, and at least 10° active extension of the thumb and any two fingers. Each participant must earn a score greater than 24 on the Mini Mental State Exam and demonstrate acceptable levels of audio and visual perception. Our sensory perception test assesses color blindness, the ability to detect basic properties of musical sounds, such as pitch, timbre, loudness, and the ability to perceive structural characteristics of the feedback such as movement of images and rhythm acceleration [13].

A participant receives 1 hour of AMRR therapy, 3 times a week for 1 month, for a total of 12 therapy training sessions. An average of 8-12 sets of 10 reaches are practiced per session depending upon the participant's ability and fatigue. Between sets the participant is able to rest, while also interact with the clinician to discuss the last set. During a therapy training session, the participant is seated at a table that is lowered or raised to provide various levels of support for the affected arm. The table also allows various target objects to be mounted and adjusted in location. Visual and audio feedback is presented on a large screen display with stereo speakers in front of the participant. While seated at the table, the participant performs a reaching task to a physical target, a cone to grasp or a large button to press, or virtual target, which requires the completion of a reach to a specified location with the assistance of audiovisual feedback. Physical and virtual target locations are presented either on the table to train supported reaches, or raised to variable heights above the table to train unsupported (against-gravity) reaches. At each height, targets can be placed at three different locations to engage different joint spaces in training.

In virtual training (with no physical target), each reach begins with a digital image appearing on the screen, which breaks apart into several minute segments of the image, referred to as particles. As the participant moves his hand towards a target location, the hand's forward movement pushes the particles back to reassemble the image and simultaneously generates a musical phrase. Any aspect of the digital feedback, however, may be turned on or off for reaching tasks to physical targets, depending on the needs of the participant, to provide mixed reality tasks and associated training. See additional file 2: Feedback generation from motion capture, for an example of feedback generated while a participant reaches within the system. The abstract feedback used within the AMRR system does not directly represent the reaching task or explicitly specify how to perform the reaching movement (e.g., the feedback does not provide a visual depiction of a trajectory to follow). Instead, movement errors cause perturbations within the interactive media that emphasize the magnitude and direction of the error (e.g., an excessively curved trajectory to the right stretches the right side of a digital image). Promoting self-assessment through non-prescriptive feedback increases the degree of problem solving by the participant and encourages the development of independent movement strategies [14,15]. The abstract feedback also recontextualizes the reaching task into performance of the interactive narrative (image completion and music generation), temporarily shifts focus away from exclusively physical action (and consequences of impaired movement) and can direct the participant's attention to a manageable number of specific aspects of his performance (e.g., by increasing sensitivity of feedback mapped to trajectory error) while deemphasizing others (e.g., by turning off feedback for excessive torso compensation).

The same abstract representation is applied across different reaching tasks (reach, reach to press, reach to grasp) and various target locations in three-dimensional space, as viewed in additional file 3: System adaptation. Thus the abstract media-based feedback provided by the AMRR system is designed to support generalization or the extent to which one training scenario transfers to other scenarios, by providing consistent feedback components on the same kinematic attributes across tasks (e.g., hand speed always controls the rhythm of the musical progression), and by encouraging the participant to identify key invariants of the movement (e.g., a pattern of acceleration and deceleration of rhythm caused by hand speed) across different reaching scenarios [16,17].

### AMRR Design Methodology

#### Representation of action and method for quantification

**Figure 3:**
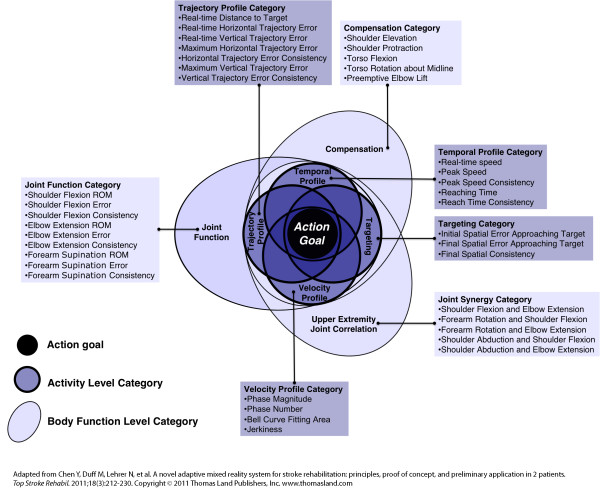
**Representation of a reach and grasp action**. Kinematic parameters are listed within seven categories: 4 activity level categories (dark background) and 3 body function level categories (light background).

The AMRR system utilizes an action representation, which is necessary for simplifying the reach and grasp task into a manageable number of measurable kinematic features. Kinematic parameters are grouped into two organizing levels: activity level and body function level categories, and seven constituting sub-categories: four within activity and three within body function, presented in Figure 3 and as detailed in [12]. The action representation is populated by key kinematic attributes that quantify the stroke survivor's performance with respect to each category of movement. Overlap between categories in the action representation indicates the potential amount of correlation among kinematic parameters. Placement relates to influence on task completion: sub-categories located close to the center of the representation have greater influence on goal completion. Each kinematic attribute requires an objective and reproducible method for quantitative measurement to be used for evaluation and feedback generation.

**Figure 4:**
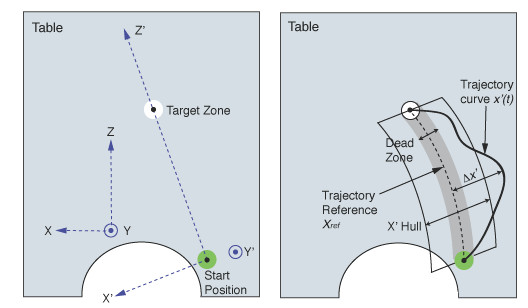
**Example of trajectory evaluation for feedback generation**. **x'(t) **is the horizontal hand trajectory (measured in cm) along the **X' **direction. **X_ref _**is the trajectory reference, from an average across non-impaired subject trajectories. The dead zone is the bandwidth for non-impaired subject variation. Trajectory deviation Δ**x' **within this zone is zero. Feedback on trajectory deviation increases or decreases exponentially as the hand moves farther away from the dead zone toward the right or left. The rate of change in trajectory deviation is controlled by the adjustable size of the hull. The wider the hull, the slower the rate of deviation change, resulting in a less sensitive feedback bandwidth. Size of the hull is adjusted by the clinician depending upon the needs of the participant.

From the three-dimensional positions of the markers worn by the participant, pertinent motion features are derived and used to compute all kinematic attributes. The quantified evaluation of these kinematic attributes is based upon four types of profile references: (a) trajectory reference, (b) velocity reference, (c) joint angle reference and (d) torso/shoulder movement reference. Each type of reference profile is derived from reaching tasks performed to the target locations trained within the AMRR system by multiple unimpaired subjects. These reference values, which include upper and lower bounds to account for variation characteristic of unimpaired movement, are scaled to each stroke participant undergoing training by performing a calibration at the initial resting position and at the final reaching position at the target. Calibrations are performed with assistance from the clinician to ensure that optimal initial and final reaching postures are recorded, from which the end-point position and joint angles are extracted and stored for reference. Real-time comparisons are made between the participant's observed movement and these scaled, unimpaired reference values. Therefore, in the context of the AMRR system, feedback communicating "inefficient movement" is provided when the participant deviates from these scaled unimpaired references, beyond a bandwidth determined by the clinician. Figure 4 presents an example of how magnitude and direction of error is calculated for feedback generation during a participant's performance of a curved trajectory.

Activity level kinematic features (see Figure 3) are extracted from the participant's end-point movement, monitored from the marker set worn on the back of the hand of the affected arm. These kinematic features, which describe the end-point's temporal and spatial behavior during a reach and grasp action, are grouped into four activity level categories: temporal profile, trajectory profile, targeting, and velocity profile. Body function kinematic features (see Figure 3) are extracted from the participant's movement of the forearm, elbow, shoulder and torso to describe the function of relevant body structures during a reach and grasp action. Body function features are grouped into three overarching categories: compensation, joint function, and upper extremity joint correlation. Monitoring these aspects of movement is crucial to determining the extent of behavioral deficit or recovery of each stroke survivor. All kinematic features and corresponding definitions for quantification within the AMRR system are summarized in Table 1. Quantification of kinematic attributes within the representation of action provides detailed information on movement performance for generation of the interactive media-based feedback.

**Table 1:** Kinematic features and corresponding definitions for quantification

Temporal profile	
*End-point speed*	The instantaneous speed at which the endpoint is moving.

*Reaching lime*	The time duration from the initiation of movement until a reach is successfully completed. A reach is completed when the end-point reaches a specified distance from the target, the end-point velocity decreases below 5% of the maximum velocity, and the hand activates a sufficient number of sensors on the force-sensing target object (if a physical target is present).

*Speed range*	The maximum speed of the end-point (within a reach) while moving towards the target from the starting position.

*Speed consistency measure*	The average variation of the maximum speed (within a reach) over a set of ten reaches.

*Reaching time consistency*	The average variation of the maximum reaching time (within a reach) over a set of ten reaches.

**Trajectory Profile**	

*Real-time trajectory error*	Real-time deviation of the end-point that is greater in magnitude than the maximum horizontal and vertical deviations within range of unimpaired variation, calculated as a function of the end-point's percentage completion of the reach.

*Maximum trajectory errors*	Largest magnitude values among the *real-time trajectory errors *within a single reach.

*Trajectory consistency*	Measurement of how trajectories vary over several reaches using a profile variation function [28].

**Targeting**	

*Target acquisition*	The binary indicator of finishing the task, achieved when the end-point reaches a specified distance from the target, the end-point velocity decreases below 5% of the maximum velocity, and the hand activates a sufficient number of sensors on the force-sensing target object (if a physical target is present).

*Initial spatial error approaching target*	The Euclidian distance between the hand position (x, y, z)_hand _and reference curve position (x, y, z)_ref _measured at the first time the velocity decreases to 5% of the velocity peak, where (x, y, x)_ref _is the reference of the hand position for grasping the target obtained from adjusted unimpaired reaching profiles.

*Final spatial error approaching the target*	The Euclidian distance between the hand position (x, y, z)_hand _and reference curve position (x, y, z)_ref _at the end of movement, where (x, y, z)_ref _is the reference of the hand position for grasping the target that is obtained during calibration.

*Final spatial consistency*	Used to measure variation of final spatial error across several trials, and is computed as the square root of summation of the ending point variances along the x-y-z directions for a set of ten trials.

**Velocity Profile**	

*Additional phase number*	The first phase is identified as the initiai prominent acceleration and deceleration by the end-point, and an additional phase is defined as a local minimum in the velocity profile beyond the initial phase. The *additional phase number *counts the number of phases that occurred beyond the first phase before reach completion.

*Phase magnitude*	Compares the size of separate phases within one reach, and is calculated as the ratio between distance traveled after the peak of first phase (during deceleration) and the distance over the entire deceleration of the reach [36]. Only the deceleration part of the first phase is examined because this portion of a reach is where the most adjustments tend to occur.

*Bell curve fitting error*	Compares the shape of the decelerating portion of the velocity profile to a Gaussian curve by measuring the total amount of area difference between the two curves.

*Jerkiness*	Measure of the velocity profile's smoothness, and is computed as the integral of the squared third derivative of end-point position [37].

**Compensation**	All compensation measures are computed as a function of the end-point's distance to target because the extent of allowable compensation varies throughout the reach [38].

*Torso flexion*	Compares the flexion of the torso relative to the non-impaired subjects' torso forward angular profile, adjusted to participant-specific start and end reference angles determined by a clinician during calibration.

*Torso rotation*	Compares the rotation of the torso relative to the non-impaired subjects' torso rotation angular profile, adjusted to participant-specific start and end reference angles determined by a clinician during calibration.

*Shoulder elevation*	Compares the elevation of the shoulder relative to the non-impaired subjects' shoulder elevation profile, adjusted to participant-specific start and end reference angles determined by a clinician during calibration.

*Shoulder protraction*	Compares the protraction of the shoulder relative to the non-impaired subjects' shoulder protraction profile, adjusted to participant-specific start and end reference angles determined by a clinician during calibration.

*Pre-emptive elbow lift*	Computed as the difference between current elbow position and the elbow position during rest calibration. Elbow lifting is only examined at the beginning of the reach as a predictive measure of initiation of the movement through compensatory strategies.

**Joint Function**	Joint angles of the shoulder, elbow and forearm are evaluated based on the following measures

*Range of motion *(ROM)	The difference in angle from the initiation to the completion of the movement.

*ROM error*	The difference between the ROM of an observed reach and the reference ROM obtained during the assisted calibration reach.

*Real-time error*	The maximum error between the observed joint angle curve during a reach and the reference curve derived from non-impaired reaching data that is scaled to the start and end reference angle of each participant.

*Consistency of the angular profile*	The average variation between angular profiles within a set often reaches.

**Upper extremity joint correlation category**	Measures synergy of two different joints moving in a linked manner, computed using the standard mathematical cross-correlation function of two angles over the duration of a reach for each pair listed below. May be compared to non-impaired upper extremity joint correlations for evaluation [39].

*Shoulder flexion and elbow extension*	Measured cross-correlation between shoulder flexion and elbow extension

*Forearm rotation and shoulder flexion*	Measured cross-correlation between forearm rotation and shoulder flexion

*Forearm rotation and elbow extension*	Measured cross-correlation between forearm rotation and elbow extension

*Shoulder abduction and shoulder flexion*	Measured cross-correlation shoulder abduction and shoulder flexion

*Shoulder abduction and elbow extension*	Measured cross-correlation between shoulder abduction and elbow extension

#### Design of Interactive media-based feedback

The interactive media-based feedback of the AMRR system provides an engaging medium for intuitively communicating performance and facilitating self-assessment by the stroke survivor. While each feedback component is designed to address challenges associated with a specific movement attribute identified in the representation, all components are designed to connect as one audiovisual narrative that communicates overall performance of the action in an integrated manner. Following the structure of the action representation, feedback is provided on performance of *activity level parameters and categories *and *body function level parameters and categories*. The *integration of individual feedback components through form coherence *also reveals the interrelationships of individual parameters and relative contributions to achieving the action goal. Example activity and body function kinematic features are listed in Table 2 with a summary of corresponding feedback components and feature selection used for each feedback component's design [12].

**Table 2:** Key kinematic features with corresponding feedback components and feature selection [12] applied within feedback design

*Activity Level Kinematic Features*	*Corresponding Feedback Components*	*Primary Sensory modality*	*Interaction time structure*	*Information processing*	*Application*
Trajectory	1.Magnitude and direction of image particle movement 2.Harmonic progression 3.Summary of error	1.visual 2.audio 3.visual	1.concurrent continuous 2.concurrent continuous 3.offline terminal	1.explicit 2.implicit 3.explicit	1.online control 2.feedforward 3.feedforward

Speed	Rhythm of music	audio	concurrent continuous	implicit	feedforward

Velocity Profile	Image formation integrated with musical progression	audiovisual	concurrent continuous	extracted	feedforward

***Body Function Level Kinematic Features***					

Forearm rotation	Image rotation	visual	concurrent continuous	explicit	online control

Elbow extension	Volume and richness of orchestral sounds	audio	concurrent continuous	implicit	online control/feedforward

Torso compensation	Abrupt disruptive sound	audio	concurrent intermittent	explicit	online control

Joint correlation	Temporal relationship among feedback mappings	audiovisual	concurrent continuous	extracted	feedforward

#### Feedback on activity level parameters and categories

Feedback on activity level parameters must assist with the movement challenges that most significantly impede the efficient performance and completion of a reaching task. Correspondingly, feedback components reflecting activity level parameters are the most detailed and prominent audiovisual elements within the AMRR feedback.

##### Activity Level Category: Trajectory profile

*Movement Challenge*: Many stroke survivors have difficulty planning and executing a linear trajectory while efficiently completing a reaching movement to a target, especially without visually monitoring movement of the affected hand [18].

*Feedback Components*: The animated formation of an image from particles, depicted with an emphasis on visual linear perspective, describes the end-point's progress to the target while encouraging a linear trajectory throughout the movement. As the participant reaches, his end-point's decreasing distance to the target "pushes" the particles back to ultimately re-form the image when the target is reached. As the expanded particles come together, the shrinking size of the image communicates distance relative to the target. The shape of the overall image is maintained by the end-point's trajectory shape: excessive end-point movements in either the horizontal or vertical directions cause particles to sway in the direction of deviation, which distorts the image by stretching it. Magnitude of deviation is communicated by how far the particles are stretched, and direction of deviation is communicated by which side of the image is affected (e.g., top, bottom, right, left, or combination thereof). To reduce the distortion of the image, the participant must adjust his end-point in the direction opposite of the image stretch. See additional file 4: Visual feedback communicating trajectory, which depicts the visual feedback generated first by a reach with efficient trajectory, followed by a reach with horizontal trajectory deviation that causes a large distortion on the right side of the image.

Formation of the image, as the most prominent and explicit stream among the feedback mappings, not only provides a continuous frame of reference for trajectory distance and shape but also communicates progress towards achieving the goal of the completed image. Furthermore, by using visual information on the screen to complete the action, and thus not simultaneously focusing visually on his hand, the participant reduces reliance on visual monitoring of his end-point.

*Principles Applied*: *Visual *feedback is best suited for communicating three-dimensional spatial information. Particle movement is directly linked to end-point movement in order to *explicitly *describe the end-point's spatial deviation from or progress towards achieving an efficient trajectory to the target. The feedback is delivered *concurrent *to action and *continuously *to allow the participant to observe movement of his end-point by monitoring formation of the image, and when needed, apply this information for *online control *of his movement to adjust for vertical or horizontal deviations.

*Movement Challenge*: Sometimes stroke survivors are unable to utilize online information during task execution to develop a movement strategy, and require feedforward mechanisms to assist with planning proceeding movements.

*Feedback Components*: A static visual summary communicates overall maximum trajectory deviation after each reach is completed to facilitate memory of real-time trajectory error. The summary presents a series of red bars. Their location on the screen (e.g., high, low, left, right, or combinations thereof) represents where error occurred in terms of vertical and horizontal coordinates (along the x, y axes respectively). Visual perspective is used to communicate the distance at which error occurred (along the z axis) through spatial depth. A deviation occurring in the beginning of the movement appears closer to the viewer in perspective space, while deviations that occur later appear further away. The number of red bars conveys the magnitude of trajectory error. See the inefficient reach presented in additional file 4: Visual feedback communicating trajectory, for an example visual summary indicating horizontal trajectory error following the completion of the image. Trajectory deviation is summarized from rest position until the hand's entrance into the target zone (an adjustable area surrounding the target that determines task completion), excluding the fine adjustment phase, as it likely does not contribute to feedforward planning of the reaching trajectory [19].

*Principles Applied*: *Visual *perspective is used to communicate the reaching distance as spatial depth. The summary provides an abbreviated history of the continuous particle movement by *explicitly *illustrating the magnitude (number of bars) and direction (location on screen) of trajectory errors. Presenting an *offline terminal *visual summary allows the participant to make an overall comparison of timing, location and magnitude of his trajectory deviations within the context of the entire reach. This display may also facilitate the *implicit processing *of the connection to memory of performance on other aspects of movement (e.g., the participant remembers hearing a shoulder compensation sound indicator in the beginning of the reach, and also sees red error bars on the top of the screen within the summary). Connecting real-time movement to *offline *contemplation can inform *feedforward *planning of successive movements.

##### Activity Level Category: Temporal profile

*Movement Challenge*: From the volitional initiation of movement until the completion of the reaching task, stroke survivors often have difficulty planning and controlling acceleration, trajectory speed, and deceleration of their movement across a defined space. This challenge makes relearning efficient movement plans difficult.

*Feedback Components*: The musical phrase generated by the participant's movement is designed to help monitor and plan the timing of movement, as well as encourage completion of the action goal. The end-point's distance to the target controls the sequence of chords of the musical phrase. The reach is divided into four sections with different musical chords played for each. The sequence of chords follows a traditional musical pattern (with some randomized variation to avoid repetitiveness) that underlies many popular songs and is thus more likely to be familiar to the participant. The participant may intuitively associate each part of the reach (early, middle, late) with a corresponding part of a musical sequence and be motivated to finish the reaching task to complete a familiar audio composition. If the end-point deviates from an efficient trajectory towards the target, the musical chords detune for the duration of deviation to place in time the occurrence of the deviation (whereas the spatial information of the deviation is communicated by the image stretching). See additional file 5: Audiovisual feedback communicating trajectory and speed, in which an efficient reach is followed by a reach with detuning as a result of trajectory deviation. Note how the addition of sound can be used to facilitate awareness of the timing of error, while the visuals accentuate error magnitude and direction.

End-point speed is mapped to the rhythm of the musical phrase. The participant's movement speed results in a "rhythmic shape" (change of rhythm over time) that most strongly encodes the end-point's acceleration during reach initiation, the deceleration when approaching the target, and the overall range of speed. In additional file 5, compare the sonic profile of the last slow reach to the sonic profile of the comparatively faster first reach, which has a noticeable acceleration/deceleration pattern and desired velocity peak. Memory of the resultant rhythmic shape (i.e., which rhythmic pattern is associated with the best reaching results) can assist the participant to develop and internalize a representation of end-point speed that helps plan his performance.

*Principles Applied*: Audio feedback is best suited for communicating temporal movement aspects. Musical feedback is controlled by the end-point's speed and distance, and communicates the end-point's *concurrent *progress towards the target in a *continuous *manner. In accompaniment to explicit visual monitoring of the image formation, the audio feedback communicates changes within the end-point's temporal activity and encourages *implicit *information processing of the rhythm as a singular, remembered form (i.e., memory of the rhythmic shape). Memory of the musical phrase supports *feedforward *mechanisms for planning future movements and facilitates comparison across multiple reaches (e.g., speed consistency of reaches within a set). The detuning of the harmonic progression adds a timestamp to the visual stretching of the image to assist *feed-forward *planning.

##### Activity Level Category: Velocity Profile

*Movement Challenge*: Many stroke survivors do not exhibit a bell-shaped velocity profile characteristic of unimpaired reaching movements as a result of difficulties with timing and executing an efficient trajectory.

**Figure 5:**
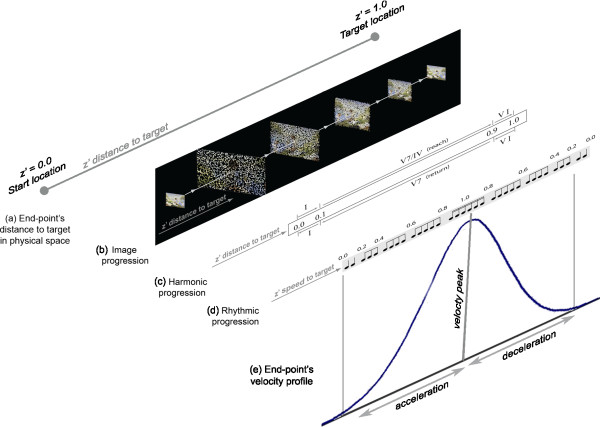
**Three parallel feedback streams communicate the spatial and temporal behavior of the end-point**. The z' distance to target (a) is the shortest physical distance the end-point must travel from rest position (z' = 0) to reach the target (z' = 1). Based on distance traveled, the end-point's location along z' prime controls the (b) size of the image formation (image progression) as well as which (c) chords are played (harmonic progression). The rhythmic shape or (d) rhythmic progression is controlled by the end-point's speed. These simultaneous feedback streams can form an integrated descriptor of the (e) velocity profile.

*Feedback Components*: Simultaneous feedback streams describing the participant's end-point behavior can help the participant in relating the temporal and spatial aspects of his reach. The acceleration/deceleration pattern communicated by the rhythmic shape of music assists the participant in understanding speed modulation. The shrinking size of the image and harmonic progression communicate his distance and overall timing to reach the target. Coupling these simultaneous mappings allows for changes in speed to be connected to distance and facilitate the development of an integrated space-time plan. Figure 5a-e illustrates how (a) the end-point’s progress to the target in the physical space is described by the (b) image progression, (c) harmonic progression, and (d) rhythmic progression, which communicate (e) velocity profile as an extracted, integrated descriptor of the end-point's distance, direction, and speed towards the target.

*Principles Applied*: As a complex aspect of movement, the velocity profile cannot be effectively expressed as a singular feedback component, for a singular mapping would not allow the participant to determine which aspect of movement (speed, direction, and/or distance) requires adjustment that generalizes to multiple types of reaching tasks. Therefore, feedback on the velocity profile is observed through extracted information processing, in which the participant integrates information from both visual and audio streams reflecting directed distance and speed. Form integration of relevant audio and visual mappings into a unified velocity profile is encouraged by feedback feature selection: all involved components are *concurrent *and *continuous *mappings of end-point movement [12]. *Extracted *information processing of the velocity profile may facilitate *feedforward *planning of acceleration and deceleration patterns along a given reaching distance to a target.

## Feedback on body function level parameters and categories

Body function level feedback must assist with challenges impeding the relearning of premorbid movement patterns of specific body structures relevant to reach and grasp performance. Feedback on body function parameters is coarse and discrete, thereby temporarily directing attention to specific body structures without distracting from completing the action goal. When a clinician must focus training on regaining functional independence primarily through teaching compensatory mechanisms, training for improved body function may not be appropriate [20,21]. Accordingly, in the AMRR system, body function mappings can be independently toggled on or off at the discretion of the clinician. Examples of each of the following feedback components may be found in additional file 6: Forearm rotation, elbow extension, and compensation.

### Body Function Level Category: Joint function

*Movement Challenge*: Many stroke survivors cannot achieve the appropriate timing or range of forearm pronation to complete a reach and grasp task.

*Feedback Components*: Feedback on forearm rotation is related to end-point activity by controlling the angle of image orientation while particles come together to form the image. Image rotation is controlled by the difference between reference forearm orientation angles at a given distance to the target and the observed forearm orientation. Excessive supination causes clockwise rotation, while excessive pronation causes counter-clockwise image rotation. The size of the rotated image communicates where forearm rotation error occurred relative to the distance from the target.

*Principles Applied*: *Visual *feedback communicates forearm spatial orientation in an *explicit *manner, which provides *concurrent*, *continuous *information throughout the reach to assist the participant's *online control*.

*Movement Challenge*: Many stroke survivors have difficulty with sufficiently extending the elbow and appropriately timing elbow extension.

*Feedback Components*: Percentage of elbow extension is mapped to a musical sequence performed by a digitized orchestra. In additional file 6 listen for the orchestral sound increasing in volume, range of pitch, and harmonic richness as the percentage of elbow extension increases and the image completes.

*Principles Applied*: Communication of elbow extension is provided as *audio *feedback to assist with timing of movement. Percentage elbow extension is mapped in a *continuous *manner, which communicates changes in magnitude of extension within a reach. Elbow extension feedback is provided *concurrently *to movement and may be used for *online control *and/or memory of its temporal relationship to other mappings can be used for *feedforward *planning. Underscoring achievement with an increasingly rich orchestral sound is a standard technique of film scoring [22]. Through *implicit *information processing, memory of successful reaches may be compared with the orchestral quality of other reaches within a set.

### Body Function Level Category: Shoulder and Torso Compensation

*Movement Challenge*: To compensate for lack of extension during a reach, many stroke survivors use excessive shoulder movement (elevation and/or protraction) and excessive torso movement (flexion and/or rotation).

*Feedback Components*: Compensatory movements are signaled by distinctive sounds that can interrupt the musical phrase (foreground) generated by end-point movement. Excessive shoulder compensation during a reach causes a cymbal sound while excessive torso compensation causes a crackling sound. These compensation indicators may be enabled individually or simultaneously, and are activated when the participant moves beyond an acceptable range of movement. Because these sounds do not combine well with the musical foreground in terms of harmony, timbre and rhythm, they draw the participant's attention to body structures exhibiting inefficient movement strategies.

*Principles Applied*: Unique audio feedback indicators provide *explicit *indication of either shoulder or torso compensation *concurrent *to action for *online control *and correction of error. These *auditory *cues allow for *intermittent *monitoring of specific body functions amidst continuous end-point monitoring. As audio indicators, *implicit *information processing of these sounds caused by compensatory movements may be integrated into memory of the overall musical phrase and overall performance to assist with *feedforward *planning.

### Body Function Level Category: Upper extremity joint correlation

*Movement Challenge*: Lack of intersegmental joint coordination while reaching for an object in three-dimensional space often results in inappropriate timing of movement at each joint.

*Feedback Components*: The relationship among different audio mappings connects individual joint function to end-point behavior. During a successful reach, as the target is approached, the rhythmic progression driven by the end-point decreases in speed, and the volume and richness of orchestral strings mapped to elbow extension increases. The peak of orchestral richness is reached at the completion of the grasp and is synchronous to the image completion, the music progression resolution, and the sounding of the success cue (triangle sound), as demonstrated in the first reach in additional file 7: Joint Correlation Examples. The orchestral sound and foreground music follow the same harmonic progression, which facilitates their integration. The memory of this desired synchronization assists the learning of a coordination schema for elbow extension in relation to end-point trajectory, velocity, and goal completion. Other body function level feedback events, such as the compensation indicator sounds, can also be placed in time along the course of the reach relative to the rhythmic and harmonic musical progression driven by the end-point, and/or related to the lack of orchestral sound. For example, in the second reach of additional file 7, the shoulder compensation sound is followed by image stretching and the detuning sound, indicating that early shoulder compensation was followed by later deviation of the end-point trajectory. Through experience with the feedback over time, memory of this relationship may occur as an *extracted *information process by the user.

*Principles Applied*: Music (*audio*) is a very effective communicator of temporal relationships between separate but parallel events [23]. Multiple relationships between musical lines (e.g., foreground melody versus background accompaniment) and individual sounds (e.g., percussive sounds related to body function) can be easily observed by listeners *concurrently *to their movement, and remembered for *extracted *processing after the event [24,25]. The intuitive memorization of the musical relationships can facilitate *feedforward *planning [26,27]. *Continuous *feedback streams that should be correlated are given different individual characteristics (e.g. timbre, rhythm) but follow a similar harmonic progression. *Intermittent *feedback indicators are used that can be related to *continuous *detailed feedback yet can still be identified as separate events.

## Integrating individual feedback components through form coherence

The connection between feedback components and corresponding movement components must be intuitive and easily perceived. Simplifying training by only offering feedback on one element at a time is not an optimal solution. Some stroke survivors cannot connect individually learned aspects of performance into a complete, generalizable, sustainable strategy [20]. The higher-level organization of the feedback must facilitate integration of multiple media components into one coherent media narrative [13].

As shown in the previous section, the AMRR system facilitates integration of multiple media streams through (a) appropriate selection of feature choices for feedback mappings (e.g., audio for time, visuals for space), (b) compositional strategies that integrate closely related streams (e.g., time and space elements of velocity profile), and (c) parallel action and feedback narratives (e.g., successful completion of the physical action goal completes the interactive media narrative). These three strategies establish formal coherence between action and media.

## Adaptive training methodology

The AMRR system is highly adaptable to maintain an appropriate level of challenge and engagement based on a participant's impairment and progress. Numerous combinations of mediated feedback and task types allow for a wide variety of training scenarios for each participant.

### Adaptable components of the AMRR system

The primary components of the AMRR system that may be adapted include target type and location, selection and sensitivity of media components, and training environment. Three task types are trained within the AMRR system: reach, reach to push a button, and reach to grasp a cone. Different task types may be applied for focused training of specific aspects of movement, such as encouraging increased speed by using a button instead of a cone as the target. Various target locations engage different joint spaces in training. The four primary target locations used within the AMRR system include supported (on the table) ipsilateral (SI), supported lateral (SL), against-gravity (elevated from table) ipsilateral (AGI), and against-gravity lateral (AGL), listed in order of complexity of joint space utilized.

Training is conducted in sets of ten reaches. Task type and feedback may be adjusted after each set. The clinician may guide the training focus for each set by selecting which media components to use. The clinician's choices also control the level of feedback complexity provided to the participant. Furthermore the sensitivity of each enabled feedback component may be independently adjusted, which allows the clinician to set the appropriate level of difficulty for each movement attribute. A hull is the adjustable amount of error in a participant's movement that the system will tolerate before giving feedback showing error (Figure 4). Adjustable, three-dimensional hulls control the feedback sensitivity for trajectory, joint function, and compensation [28]. The peak rhythm of the musical phrase may also be scaled to a desired maximum hand speed determined by the clinician.

Different training environments may be used within the AMRR system by controlling the amount of media-based feedback (virtual) and task (physical) components applied. The AMRR system provides for gradual transitions between virtual and physical training by using three types of training environments: physical (provides no media-based feedback while reaching to a physical target), hybrid (provides combinations of media-based feedback while reaching to a physical target) and virtual (requires interaction with only media-based feedback and no physical target). Multiple available variants (gradations) of these environments enable the clinician to shift training on a continuum towards a more virtual (for recontextualization [12]) or more physical (for reduced or no feedback guidance) environment depending upon the participant's needs and stage of training.

### Overview of adaptive training

Before training with the AMRR system begins, a participant is evaluated by the evaluating clinician and an attending physician to determine and rank the movement aspects requiring focused training. This collection of movement aspects from the preliminary assessment is called the impairment profile and may be used to guide the adaptive training. For each set of ten reaches, the treatment clinician typically chooses one or two limitations from the limitation profile on which to focus. Assisted by the media specialist, the clinician may then select the appropriate task components (target type and location), media-based feedback components (which feedback components to enable and respective sensitivities) and type of training environment (virtual, hybrid, physical) to utilize for training. Sequential modifications of these training components and movement aspects form the adaptive training. After each set, the system's quantitative evaluation, coupled with the clinician's direct observation, inform the adaptation decisions for the proceeding set. The AMRR system's ability to adapt along several dimensions throughout training allows for a customized training experience for each participant. For more description on the dynamics of training in application please refer to Chen et al, in which two example patient case studies are presented [16].

The study protocol has been reviewed and approved by the Institutional Review Board at Arizona State University. All participants signed a consent statement and authorization forms. Copies of these signed forms are available for review by the Editor-in-Chief of this journal upon request. No participant's facial identity is revealed within the media associated with this manuscript.

## Methods for Outcome measurement & Data analysis

Training using the AMRR system is evaluated using changes in kinematic performance and standard clinical assessments as outcomes, each of which are measured prior to and after four weeks of training. Kinematic performance is measured from 10 reaches, unassisted by feedback, to each of the 4 trained target locations for a total of 40 reaches. Full clinical assessments include the Motor Activity Log (MAL), the Stroke Impact scale (SIS), the upper extremity portion of the Fugl-Meyer Assessment (FMA), and the Wolf Motor Function Test (WMFT) [29-32].

Pre and post values are presented here from the WMFT and the upper extremity motor function portion of the Fugl-Meyer Assessment. The WMFT consists of a series of functional tasks relevant to activities of daily living that are timed and rated for quality by the evaluating clinician [32]. The FAS rating scale ranges from 0 = no attempt to 5 = normal movement. The rating scale for the Fugl-Meyer Assessment ranges from 0 = cannot perform to 2 = performs completely [31].

Supportive kinematic data are provided within this paper to demonstrate successful application of the system's feedback components to achieve focused and integrated improvements in three representative participants with different movement impairments. The raw pre and post training measurements of 12 kinematic features related to activity and body function movement aspects trained using the AMRR feedback are presented with respect to the three participants' training needs. Pre and post kinematic performance were analyzed using the Wilcoxon rank-sum Test due to the small sample number of reaches [33]. Statistical significance was measured at α = 0.05 and α = 0.01, and to account for multiple comparisons across 12 kinematic parameters, at α = 0.004 and α = 0.0008.

In addition to kinematic measures and standard clinical assessments for evaluation, methods are being developed to better gauge the added value of AMRR feedback to rehabilitation training on the level of individual reaches (*trials*). Individual trials are compared in sequential pairs to evaluate the correlation between observable feedback in one trial and improved performance of kinematic variables in the trial immediately following. Percentage improvement in performance of a specific kinematic variable after the presence of feedback was calculated as follows:

1. A trial *k *was selected if performance of a kinematic variable *deviated *beyond a threshold determined from movement references measured from unimpaired individuals.

2. Within these selected trials, if error feedback was triggered, the trial *k *was classified as *with feedback *(FB). If the error feedback was not triggered (due to low feedback sensitivity) or the feedback component was turned off for that trial, the trial *k *was classified as *no feedback *(NFB).

3. For the two resulting sets of FB and NFB trials, the mean performance of all initial trials *k *(*pk mean*) was compared to the mean performance of all immediately following trials *k+1 *(*pk+1 mean*) by calculating the percentage change:

4. The difference in percentage improvement between the NFB and FB sets of trials was calculated:

## Supportive Data

Results are presented from the pre and post evaluations of three stroke survivors who have each trained with the AMRR system for a period of 12 weeks. Table 3 lists demographic information and lesion type of the three participant stroke survivors, each of whom was right-sided hemiparetic. Based on the pre-training evaluation, a unique impairment training profile was determined by the physical therapist and attending physician for each participant. Table 4 lists the ranked movement aspects of each participant's impairment profile. For each set during training, the clinician determines the movement focus and selects the appropriate feedback components and sensitivity to use. Table 4 includes the corresponding distribution of resultant focused training on relevant movement aspects, presented as percentages of total sets completed within the 12 week training period.

**Table 3:** Participant demographics and lesion type

	Age	Months post-stroke	Sex	Lesion
***Participant 1***	76	6	Female	left basal ganglia & periventricular white matter infarct

***Participant 2***	74	7	Male	left-sided middle cerebral artery infarct

***Participant 3***	66	6	Male	multifocal embolic left hemispheric cerebral infarctions

**Table 4:** Participant baseline impairment and resultant training distribution

	Rated Overall Impairment	Ranked Movement Aspects of Impairment Profile	Related Movement Features	% focused training
***Participant 1***	Mild	1. Torso and shoulder compensation	Shoulder compensation	35.09
		2 Inconsistency of elbow extension	Peak speed	15.00
		3. Insufficient elbow extension	Torso compensation	12.57
		4. Trajectory	Targeting	9.65
		5. Joint Synchrony	Others	9.52
			Elbow extension	9.11
			Trajectory	9.06

***Participant 2***	Mild to moderate	1. Insufficient elbow extension	Torso compensation	28.9
		2. Insufficient shoulder flexion	Elbow extension	23.2
		3. Insufficient speed	Others	20.2
		4. Slow initiation of movement	Trajectory	12.5
		5. Torso compensation	Peak speed	8.1
			Velocity bellness	7.1

***Participant 3***	Moderate	1. Insufficient elbow extension	Torso compensation	21.5
		2. Insufficient shoulder range of motion	Others	20.6
		3. Shoulder and torso compensation	Trajectory	19.1
		4. Ataxia	Elbow extension	18.2
		5. Targeting	Velocity bellness	12.7
			Joint correlation	7.9

**Figure 6:**
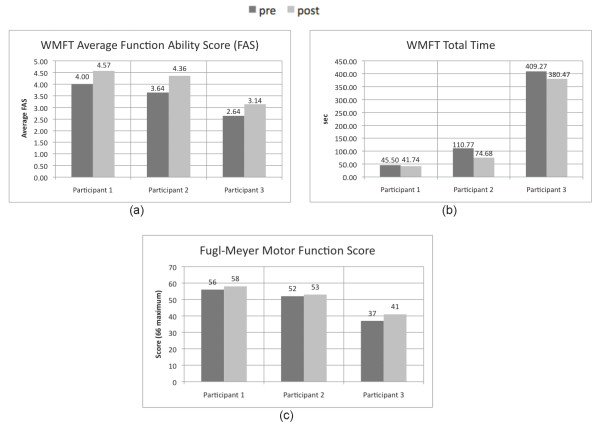
**Changes in the Wolf Motor Function (WMFT) and Fugl-Meyer Assessment**. (a) WMFT average Functional Ability Score (FAS), (b) WMFT total time, and (c) Fugl-Meyer Motor Function Assessment score

Figure 6 displays the results from the Wolf Motor Function Test (WMFT) and motor function section of the Fugl-Meyer Assessment (FMA) performed prior to and after training. All three participants increased their average FAS, indicating an increased quality of movement as rated by the evaluation therapist, as well as decreased the total time required for task completion among the tasks tested. Each participant also improved his or her Fugl-Meyer motor function score as well.

**Figure 7:**
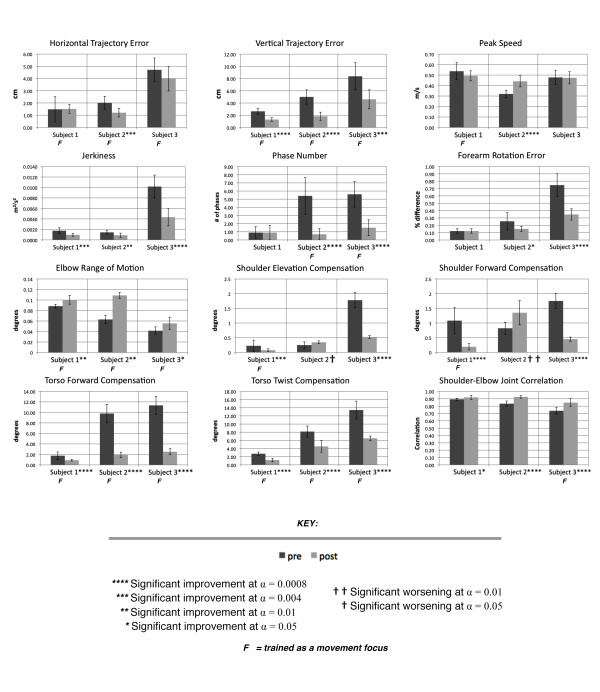
**Average changes in kinematic parameters measured prior to and following 12 sessions of training with the AMRR system**. Raw mean values are presented for the unsupported (6-inches above the table surface) cone target located at the participant's midline, which is one of four target locations evaluated during the pre and post evaluation sessions.

Figure 7 presents the average changes in kinematic performance for both activity and body function level movement aspects, measured prior to and immediately following AMRR training. Measurements are taken from a set of ten reach to grasp movements, unassisted by feedback, using a cone located at the midline and elevated 6 inches off the table. The level of significant difference between the pre and post training measurements is indicated next to each participant number. Movement aspects that received training focus are indicated. Despite differences among training regimens, all 3 participants experienced significant improvement not only in movement aspects that received focused training, but also in movement aspects that were beyond training focus areas.

Among the kinematic parameters listed in Figure 7, trajectory error, shoulder compensation, torso compensation, and supination error each are mapped to concurrent feedback that is designed for explicit awareness of performance. Thus error feedback on these attributes can be utilized immediately by the participant to correct movement performance in a subsequent trial. For these attributes, Table 5 lists the difference in percentage improvement between adjacent reaches when feedback was present versus when feedback was not. Cells with bolded text indicate percentages of improvement for a kinematic parameter given its corresponding feedback mapping. For most of the variables listed, kinematic performance improved more (indicated by the positive percentage listed) following erroneous trials that triggered its corresponding feedback as compared to following trials that did not have this feedback mapping expressed. For all 3 participants, for example, forearm rotation error improved by more in trials that followed the triggered feedback mapping for forearm rotation error (i.e. image rotation) as compared to following trials with error in forearm rotation that did not trigger the feedback. Similar patterns of improvement can be seen for all 3 participants for vertical trajectory error and torso compensation, indicated by the bolded values demonstrating positive percentage improvement.

**Table 5:** Difference in percentage improvement for explicit FB versus the NFB trials for kinematic variables reflecting trajectory, forearm rotation, shoulder and torso compensation

		**Horizontal Trajectory Error *Feedback***	**Vertical trajectory Error *Feedback***	**Forearm Rotation Error** ***Feedback***	**Shoulder Compensation** ***Feedback***	**Torso Compensation** ***Feedback***
	
***Participant 1***	Horizontal Trajectory Error	**-7.7%**	-16.0%	-22.7%	7.1%	8.4%
	
	Vertical Trajectory Error	52.5%	**52.5%**	43.5%	47.4%	36.2%
	
	Forearm Rotation Error	22.4%	38.4%	**7.1%**	2.0%	44.2%
	
	Shoulder Elevation Compensation	-3.2%	20.7%	-5.9%	**-4.0%**	19.6%
	
	Shoulder Forward Compensation	13.9%	21.4%	0.8%	**9.1%**	31.5%
	
	Torso Forward Compensation	37.7%	33.6%	23.7%	-3216.0%	**39.2%**
	
	Torso Twist Compensation	18.7%	26.7%	-31.1%	2.3%	**30.3%**
	
***Participant 2***	Horizontal Trajectory Error	**10.3%**	4.8%	38.6%	24.0%	2.3%
	
	Vertical Trajectory Error	24.8%	**29.0%**	22.5%	-11.5%	38.0%
	
	Forearm Rotation Error	9.0%	25.7%	**36.4%**	23.3%	-9.3%
	
	Shoulder Elevation Compensation	-2.3%	-2.0%	-5.3%	**20.6%**	-8.8%
	
	Shoulder Forward Compensation	5.9%	3.5%	-4.7%	**-5.2%**	5.9%
	
	Torso Forward Compensation	19.1%	12.1%	11.2%	6.5%	**25.3%**
	
	Torso Twist Compensation	11.8%	27.5%	3.6%	26.3%	**16.9%**
	
***Participant 3***	Horizontal Trajectory Error	**13.0%**	1.3%	-3.1%	-184.0%	2.2%
	
	Vertical Trajectory Error	2.5%	**22.1%**	-23.8%	17.8%	11.1%
	
	Forearm Rotation Error	-0.2%	0.1%	**31.0%**	5.9%	-1.8%
	
	Shoulder Elevation Compensation	4.1%	2.1%	5.8%	**0.9%**	0.8%
	
	Shoulder Forward Compensation	0.1%	0.8%	0.6%	**27.5%**	-2.2%
	
	Torso Forward Compensation	33.7%	27.1%	27.6%	60.2%	**36.2%**
	
	Torso Twist Compensation	16.4%	6.0%	12.7%	-1.8%	**12.6%**

Table 5 also lists the difference in % improvement for feedback versus no feedback trials in the performance of a given kinematic variable following the triggering of feedback mapped to *different *kinematic variables (cells with non-bolded values). For example, for all 3 participants, torso forward compensation improved more after trials that triggered feedback on horizontal or vertical trajectory error (i.e. horizontal or vertical image stretching) than after trials that had significant torso compensation error but did not trigger image stretching.

## Discussion

Table 4 demonstrates that although a participant's impairment profile can inform training, the resultant training dynamics are highly catered to an individual's progress and therefore may deviate from the initial impairment profile. Because the clinician can dynamically adapt AMRR training to respond to the participant's needs within or across sessions, training can focus on movement aspects that deviate from those listed within the impairment profile. Furthermore, the training distribution (number of sets that are focused on each movement attribute) does not necessarily reflect a movement attribute's rank in the initial impairment profile, as indicated in Table 4.

The generalizable value of AMRR training is supported by improvements in both the WMFT FAS scores (Figure 6) and kinematic measures (Figure 7) across the three participants, since the tasks trained in reach and grasp are not the same as the tasks assessed in the WMFT FAS. Due to the small number of samples, no statistical comparisons are provided. Though Participant 3 was able to achieve the most improvement in the motor function section of the Fugl-Meyer assessment, the other two participants, rated with lower impairment levels, may have made more gains had the training period been extended beyond twelve sessions.

Table 4 and Figure 7 demonstrate that customized approaches to training can result in kinematic improvements for participants of different impairment. Though each participant experienced training with different focus distributions, each participant improved across almost all of their respective focus areas. The exceptions are Participants 1 and 3 who did not improve in horizontal trajectory, which may be related to more attention given to accuracy with respect to the target's elevation, and Participant 1 who did not increase in speed, but remained within close range of 0.5 m/s. All participants improved in multiple aspects of movement that were not designated as focus areas as well.

An example of training using feedback on correlated aspects of movement can be seen in Participants 2 and 3, who each began training with insufficient elbow extension as a top-ranked movement impairment and experienced most of his focused training in torso compensation. This example illustrates how a more direct mapping encouraging explicit information processing on torso compensation was also utilized to promote elbow extension. Multiple types of feedback (e.g. feedback encouraging explicit versus implicit processes) catered in design to communicate performance of specific aspects of movement (trajectory versus joint relationships) can allow for both targeted and integrated training of multiple movement aspects. For example, in Participant 2's training, feedback on trajectory and speed were utilized to train individual performance aspects with resulting improvements, or were utilized in parallel to focus on training velocity bellness, which also improved. Improvement in joint correlation also utilized the integration of individual intermittent mappings in the context of continuous streams of activity level feedback in all 3 participants, for example, to improve performance of both individual joint function and integrated joint correlation.

Training also accommodates focus on activity level versus body function level movement aspects. Participant 1, rated with mild impairment, was able to maintain the majority of her activity level performance while improving all of her body level aspects that related to focused training. While over half of Participant 2's training focused in torso compensation and elbow extension, he experienced significant improvements across most features presented in both body function and activity level training. Participant 2's significant worsening in shoulder compensation could potentially be addressed with longer treatment periods allowing for focused training on this parameter.

Table 5 presents increased percentage improvement in kinematic performance for most kinematic-feedback mapping pairs (bolded values) across all 3 participants. These results reveal indices that concurrent AMRR feedback designed for explicit understanding on performance of a given kinematic variable can lead to immediate self-assessment and increased correction for that targeted aspect of movement. Furthermore, the correlation between increased improvements in a kinematic parameter and the presence of feedback mapped to *different *kinematic parameters may suggest that AMRR feedback promotes integrated training; it can allow the user to derive relationships between different aspects of movement through an extraction process (i.e. gain understanding after longer term experience with multiple feedback components), such as the effect of proximal body movements on distal end-point activity. Differences among the participants' performance improvements in relation to the triggered feedback demonstrates that the effectiveness of each feedback mapping can vary by user, providing support that an adaptable feedback paradigm catering to the needs of each participant is necessary.

Future work will include analysis of relationships between other feedback mappings that encourage more implicit processes (e.g. elbow extension, joint correlation, velocity profile) and correlated performance improvements. These aspects of movement, which require longer experience with the system, must in turn be assessed across multiple trials or sessions.

The AMRR system has been used and accepted by both stroke survivors and clinicians through a series of studies that demonstrate its reliability and validity [13,16,17]. We have discussed the experiences with all stroke survivors and clinicians that have used and are currently interacting with the system, and utilize their feedback for improvement. This is a continuous process, as new users offer valuable feedback for the improvement of the system's design. Thus, we are considering the development of a more standardized qualitative evaluation of the system by the users. Additionally, feedback on grasping is under current development, as well as feedback that elicits reaction without causing tension, so that we can consider reaction time as part of our evaluation. Knowledge gained through application of the clinical system design in practice also informs the development of a home-based rehabilitation system.

Currently a home-based adaptive mixed reality rehabilitation (HAMRR) system is under development that uses the design principles of the AMRR system to allow stroke survivors to continue their treatment over multiple months of therapy at home. Multimodal sensing methods and audiovisual feedback provide concurrent and summary information on task performance to assist the stroke survivor in self-assessing his movement. Within each week of training, a computational adaptation component automatically customizes weeklong therapy sessions based on a clinician's goals for the stroke survivor. The adaptation is structured as a semi-supervised framework, using a probabilistic graphical network representation based on [34]. The system archives performance evaluations of the stroke survivor's progress for the clinician to review remotely on a weekly basis and adjust training accordingly. After the therapist reviews the stroke survivor's movement evaluation, he or she can customize parameters in the adaptation framework, such as training focus and sequence, which will further inform the automatic adaptation used within weekly training. The HAMRR system is scheduled for testing by stroke survivors in their homes in the fall of 2011.

## Conclusions

The methodology for designing a mixed reality system for stroke rehabilitation discussed in our companion paper[12] has been applied for the development of an Adaptive Mixed Reality Rehabilitation (AMRR) system for upper extremity rehabilitation of stroke survivors. The AMRR system provides evaluation and adaptable feedback tools for the clinician to promote effective customized training. From preliminary analysis of the data, we infer that the system's ability to train multiple foci together or in isolation in adaptable sequences, by utilizing appropriately designed feedback with cohesive form, can lead to functional improvement. Improvements for parameters with focused training are assumed to reflect the clinician's directed adaptation of the system to enhance the participant's concentrated practice of those aspects of movement. Body function improvements, occurring in conjunction with activity level improvements or sustained activity level performance, support the system's ability to provide integrated training towards achieving activity level recovery without fostering compensatory behavior. Feedback-performance correlations on a per trial basis suggest that AMRR explicit feedback is improving targeted aspects of performance and promoting integrated improvements across multiple aspects of movement.

## Competing interests

The authors declare that they have no competing interests.

## Authors' contributions

NL, TR, YC and SLW contributed to the concepts and of the paper. NL and YC prepared the manuscript. TR, SLW and MD provided editing and consultation. All authors read and approved the final manuscript.

## Supplementary Material

Additional file 1 **QuickTime movie**. Depicts a participant interacting with the system in the presence of a physical therapist. Click here for file

Additional file 2 **QuickTime movie**. Depicts motion capture tracking in parallel to a participant interacting with the system. Click here for file

Additional file 3 **QuickTime movie**. Depicts a participant interacting with different targets and target locations within the system. Click here for file

Additional file 4 **QuickTime movie**. Depicts the visual feedback generated by (1) an efficient reach, followed by a reach exhibiting (2) trajectory error, communicated as image stretching. The box and diamond appearing before and after each reach is a visual representation of the location of the participant’s right hand, provided to assist the participant in returning his or her hand to a consistent rest zone on the table. Click here for file

Additional file 5 **QuickTime movie**. Depicts the image formation and plays the musical phrase generated when a participant performs (1) an efficient reach, followed by (2) a reach with a large horizontal trajectory deviation to the right that generates image stretching and a detuning musical phrase, followed by (3) a slow reach without a prominent rhythmic shape. Click here for file

Additional file 6 **QuickTime movie**. (1) Depicts image rotation when a participant incorrectly times forearm rotation, (2) the orchestral accompaniment generated when a participant extends his elbow while performing an efficient reach, (3) the shoulder compensation sound generated when a participant uses excessive shoulder compensation during the reach, and (4) the torso compensation sound generated when a participant uses excessive torso compensation during the reach. Click here for file

Additional file 7 **QuickTime movie**. (1) Depicts image formation and plays the musical phrase with orchestral accompaniment generated when a participant extends his elbow while performing an efficient reach, and (2) depicts image formation, stretching, and summary of error, and plays the shoulder compensation sound amidst the musical phrase, generated when a participant uses excessive shoulder compensation during the reach, and as a result performs related trajectory deviations. Click here for file
